# 1550. Engaging Black Women on Cabotegravir LA for PrEP by Optimizing Novel Implementation Strategies (EBONI) Study: Provider Perceptions of Appropriateness of Cabotegravir LA for PrEP for Cis-and-Trans Black Women

**DOI:** 10.1093/ofid/ofad500.1385

**Published:** 2023-11-27

**Authors:** Teriya Richmond, Michael Dunn, Marye Bernard, Rimgaile Urbaityte, Kenneth Sutton, Denise Sutherland-Phillips, Alftan Dyson, Deanna Merrill, Samantha Chang, Nicole Mack, Amber Haley, Kimberley Brown, Tammeka Evans

**Affiliations:** Next Innovative Clinical Research, Houston, Texas; Midway Specialty Care Center, Tampa, Florida; Spirit Health, Memphis, Tennessee; GSK, Uxbridge, England, United Kingdom; ViiV Healthcare, Research Triangle Park, North Carolina; ViiV Healthcare, Research Triangle Park, North Carolina; ViiV Healthcare, Research Triangle Park, North Carolina; ViiV Healthcare, Research Triangle Park, North Carolina; RTI International, Research Triangle Park, North Carolina; RTI International, Research Triangle Park, North Carolina; ViiV Healthcare, Research Triangle Park, North Carolina; ViiV Healthcare, Research Triangle Park, North Carolina; ViiV Healthcare, Research Triangle Park, North Carolina

## Abstract

**Background:**

Black women comprise 55% and 46% of new HIV diagnoses among cisgender and transgender women in the United States, respectively.^1^ Research has demonstrated the superiority of long-acting cabotegravir (CAB LA) for PrEP in reducing the risk of sexually acquired HIV-1 infection compared to oral PrEP (TDF-FTC). EBONI is a phase 4 effectiveness-implementation hybrid study focused on evaluating implementation of CAB LA delivery to Black cisgender and transgender women in US Ending the HIV Epidemic (EHE) jurisdictions. Results from the baseline survey of Staff Study Participants (SSPs) are presented here.

^1^ CDC, 2021; CDC, 2019

**Methods:**

SSPs (N=65) from 14 clinics completed surveys with questions pertaining to their clinic characteristics, perceptions of populations appropriate for CAB LA, and their perceived appropriateness of CAB LA for Black women as measured by the Intervention Appropriateness Measure (IAM). The IAM uses a 5-point rating scale (1=completely disagree to 5=completely agree). One designated SSP for each clinic completed a questionnaire to assess use of PrEP at the clinic level.

**Results:**

SSP and clinic-level characteristics are described in Tables 1 and 2. Most clinics were in the South (71.4%) and were private practice (36.4%), FQHCs (18.2%), or community-based organizations (18.2%). A total of 225 Black women across all clinics used any kind of PrEP and 69 received at least one injection of CAB LA at baseline. Most SSPs (86.2%) reported patients asked about CAB LA in their clinic.

Overall, SSPs perceived Black women in their clinic as appropriate for CAB LA (IAM Mean Score=4.6). Sixty-six percent of SSPs reported there were individuals with specific demographics more appropriate for CAB LA. Of these, lesbian women, people older than 50 years of age, and heterosexual men were rated relatively lower than other demographics as appropriate by SSPs (Table 3). Having a partner living with HIV, condomless sex, and having multiple sexual partners were rated as behaviors most appropriate for CAB LA.

**Figure:**
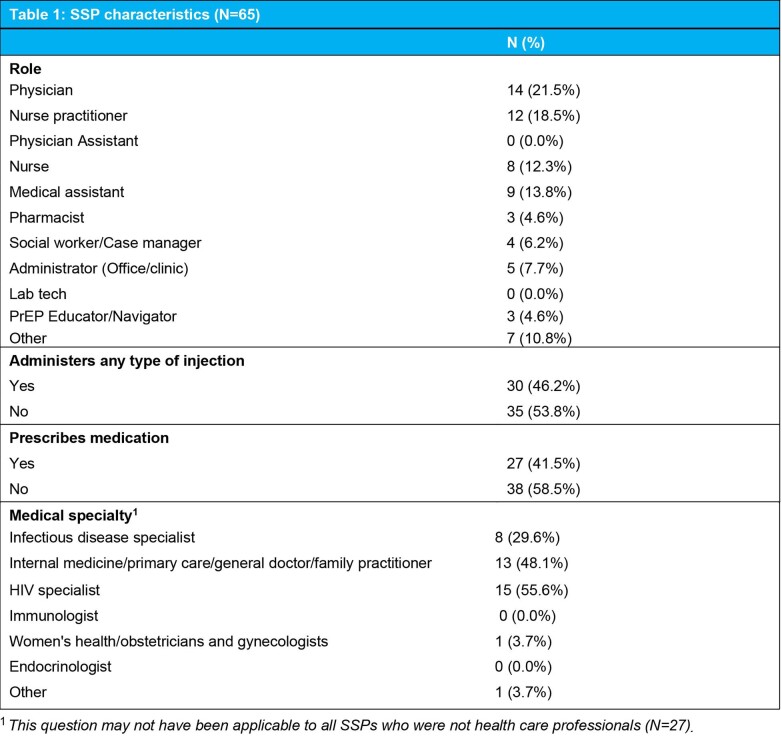

**Figure:**
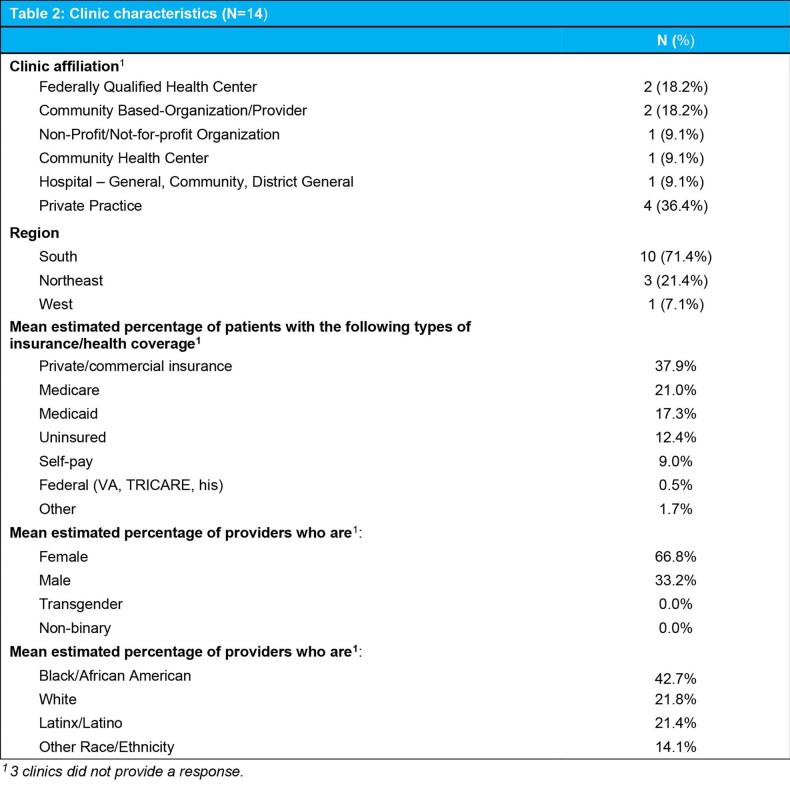

**Figure:**
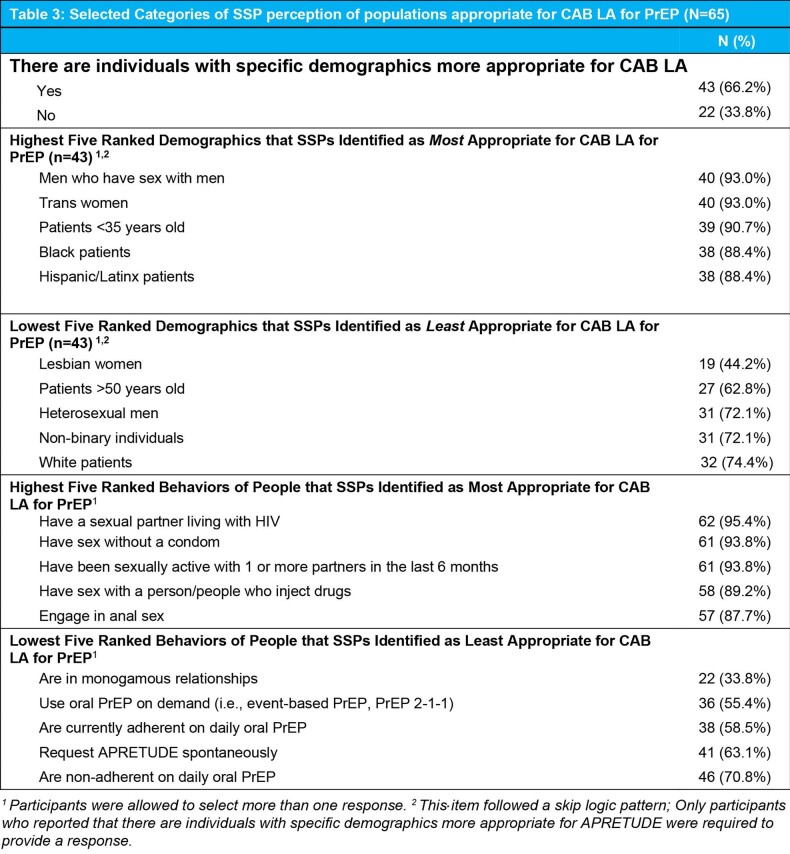

**Conclusion:**

Healthcare staff reported high levels of appropriateness of CAB LA for Black women and ranked populations and behaviors traditionally associated with PrEP as most appropriate for CAB LA. Better tools are needed to support SSPs in identifying individuals who could benefit from CAB LA.

**Disclosures:**

**Michael Dunn, MD**, Gilead: Speaker **Rimgaile Urbaityte, MSc**, GSK: Employment|GSK: Stocks/Bonds **Kenneth Sutton, MA**, ViiV Healthcare: Employment|ViiV Healthcare: Stocks/Bonds **Denise Sutherland-Phillips, MD**, ViiV Healthcare: Employment|ViiV Healthcare: Stocks/Bonds **Alftan Dyson, PharmD**, GSK: Stocks/Bonds|ViiV Healthcare: Employment **Deanna Merrill, PharmD, MBA, AAHIVP**, ViiV Healthcare: Employment|ViiV Healthcare: Stocks/Bonds **Amber Haley, PhD**, ViiV Healthcare: Former Employment **Kimberley Brown, PharmD**, J&J: Stocks/Bonds|ViiV Healthcare: Empoyment **Tammeka Evans, MoP**, ViiV Healthcare: Employment|ViiV Healthcare: Stocks/Bonds

